# Knowledge maps: a tool for online assessment with automated feedback

**DOI:** 10.1080/10872981.2018.1457394

**Published:** 2018-04-02

**Authors:** Veronica W. Ho, Peter G. Harris, Rakesh K. Kumar, Gary M. Velan

**Affiliations:** a Dept of Pathology, School of Medical Sciences, Faculty of Medicine, UNSW Sydney, Sydney, Australia; b Office of Medical Education, Faculty of Medicine, UNSW Sydney, Sydney, Australia

**Keywords:** Knowledge maps, concept maps, online, assessment, feedback, modified essay question

## Abstract

In higher education, most assessments or examinations comprise either multiple-choice items or open-ended questions such as modified essay questions (MEQs). Online concept and knowledge maps are potential tools for assessment, which might emphasize meaningful, integrated understanding of phenomena. We developed an online knowledge-mapping assessment tool, which provides automated feedback on student-submitted maps. We conducted a pilot study to investigate the potential utility of online knowledge mapping as a tool for automated assessment by comparing the scores generated by the software with manual grading of a MEQ on the same topic for a cohort of first-year medical students. In addition, an online questionnaire was used to gather students’ perceptions of the tool. Map items were highly discriminating between students of differing knowledge of the topic overall. Regression analysis showed a significant correlation between map scores and MEQ scores, and responses to the questionnaire regarding use of knowledge maps for assessment were overwhelmingly positive. These results suggest that knowledge maps provide a similar indication of students’ understanding of a topic as a MEQ, with the advantage of instant, consistent computer grading and time savings for educators. Online concept and knowledge maps could be a useful addition to the assessment repertoire in higher education.

## Introduction

Assessment and feedback are fundamental components of educational practice, from early childhood through to university and continuing professional education. Medical education is no exception. Not only do assessments help teachers to determine students’ knowledge and skill base, but assessments also affect student learning by shaping and directing further study [1,2].

In written examinations, two broad categories of questions exist: closed and open ended. The latter type can sometimes be seen as more robust, because students must generate their own answers, thereby avoiding any cueing of responses [3,4]. Some also believe that open-ended questions cover an enhanced integration of knowledge, such as interpretation of information, problem-solving skills, and critical thinking [5–7]. However, perhaps counterintuitively, the evidence does not support the argument that open-ended questions address higher-order cognitive skills better than well-written multiple-choice questions (MCQs) [8]. Furthermore, grading open-ended questions is labor intensive, particularly if students are to be given meaningful feedback that can support their learning [9,10]. This is further amplified in formative assessments, where the emphasis is on guiding learning as opposed to judging competency [11]. Some have argued that based on the cost of resources alone, MCQs might be superior to open-ended questions, particularly extended response items or modified essay questions (MEQs) [12]. Supporters of this argument might look to Schwartz and Loten [13], who administered an assessment that consisted of objective items requiring students to select an alternative from a list. Students were also asked to explain their answers. Both the answers and the explanations were scored separately. The authors found a very high correlation between students’ scores for objective items and their score for explanations, suggesting that predominantly, students arrived at the correct answer only if they used the correct reasoning [13]. This, and other evidence [12,14–19], adds weight to the argument that well-constructed closed objective items may have equivalent validity to open-ended questions.

The most frequently employed closed assessment items are MCQs. Well-constructed MCQs can test higher-order thinking, evaluating students’ ability to judge, integrate, synthesize, and apply their knowledge [20]. Compared to extended response items or MEQs, MCQs take up very little examination time, which means that a broader selection of topics can be assessed in a short period, thereby enhancing the reliability of the assessment. Well-constructed MCQs that address integrated understanding of concepts can reflect the intended learning outcomes of a curriculum of study.

An MCQ that requires the student to make a judgment, for example, a clinical scenario requiring a decision about patient management, is an example of how higher-order thinking might be addressed [12]. However, such MCQs are difficult and time-consuming to develop [21]. Effective functioning distractors must be neither trivial nor ludicrous [4], nor should they cue students to the correct answer. Functioning distractors have been defined as options selected by >5% students and chosen by lower-performing students more than higher-performing students [22], and can be difficult to write [23].

Given the difficulty of generating large banks of well-written MCQs, variations such as ‘un-cued MCQs’ and extended matching items have come into use. ‘Un-cued MCQs’ present only the stem to students, who must respond to each item by referring to a long, alphabetized list of alternative answers and entering the numerical code corresponding to the student’s choice. These lists are assumed to take too long for students to read through within the time available, thereby earning the label ‘un-cued’. Such MCQs are faster to write than traditional MCQs because there is no need to generate distractors, which means that it might be easier to accumulate a large pool of questions that can be reused. However, care must be taken to check for possible multiple correct responses [24]. ‘Un-cued MCQs’ can be scored just as quickly, accurately, and economically by computer as traditional MCQs, whilst minimizing the possibility that students might arrive at the correct answer by chance. Automated scoring not only reduces teacher workload, but also means that students can receive timely feedback. Moreover, ‘un-cued MCQs’ have been shown to differentiate between experienced and inexperienced clinicians more accurately than single best answer MCQs [4].

Extended matching questions are MCQs that present a list of 15–20 choices instead of the traditional five choices. It has been previously suggested that presenting students with 20 choices is statistically as reliable and valid as ‘un-cued MCQs’ [25]. Extended matching questions have been found to be easier to administer and cause less pretest anxiety and criticism from students than ‘un-cued MCQs’, because students are more familiar with the format [25].

### Concept and knowledge maps for assessment

A novel approach to assessment is the use of online concept and/or knowledge maps. Concept and knowledge maps are graphical representations of knowledge, and have been used in all levels of education to promote meaningful learning, critical thinking, and problem-solving skills [26–29]. They are composed of concepts (typically enclosed in boxes) and are joined together by lines or arrows and labeled with linking phrases. Characteristic features of concept and knowledge maps are a hierarchical structure (with broader concepts at the top) and cross-links (showing how ideas from different domains relate to each other [30]. Within medical education, concept and knowledge maps have been shown to help students integrate their understanding of physiological mechanisms [31,32] and pathology [33,34], and help students transfer knowledge learnt from one context to another [35].

It has been suggested that using concept and/or knowledge maps as an additional form of assessment may help to emphasize meaningful integrated learning and understanding [33,34,36]. Student-constructed maps can be manually graded, but this takes substantial time and effort [37,38]. The perceived workload implications and difficulty of marking such maps has discouraged their wider uptake as assessment items. Online automated assessment of maps could potentially overcome this obstacle, but first must prove to have the similar reliability and validity to traditional methods of assessment [39]. Concept and/or knowledge maps have the advantage of inherently emphasizing interrelationships and integration of concepts, thereby affording utility at least equal to well-constructed MCQs and MEQs, without the marking burden of the latter.

All these types of items have their strengths and weaknesses, and may be suitable for different contexts depending on the purpose of the assessment. In this report, we present a study of new technology for automated online assessment of knowledge and concept maps, which could be added to the repertoire.

## Methods

### Knowledge maps

Knowledge maps is an online concept and knowledge-mapping tool that can be used to create, edit, share, and assess maps. The tool can provide automated feedback on students’ maps in realtime, and is completely online, accessible from any modern web browser without additional plugins or applications. This tool has been described in more detail elsewhere [40], but the basic scoring mechanism uses the weighted proposition method, as developed by Chang et al [41], whereby each proposition in the teacher’s map is given a weight from 0.0 to 1.0 (i.e., more important or central propositions were given higher weighting) and these weightings are totaled. The student’s score is determined by comparing their propositions to the corresponding proposition in the teacher’s map. Correct answers are given full weight, partially matched answers given half weight, and missing answers are awarded zero. This score is then made available to the students as a percentage of the total score compared with the teacher’s map. This article focuses on a new application of the knowledge maps tool, which is known as the assessment mode.

**Figure 1.**
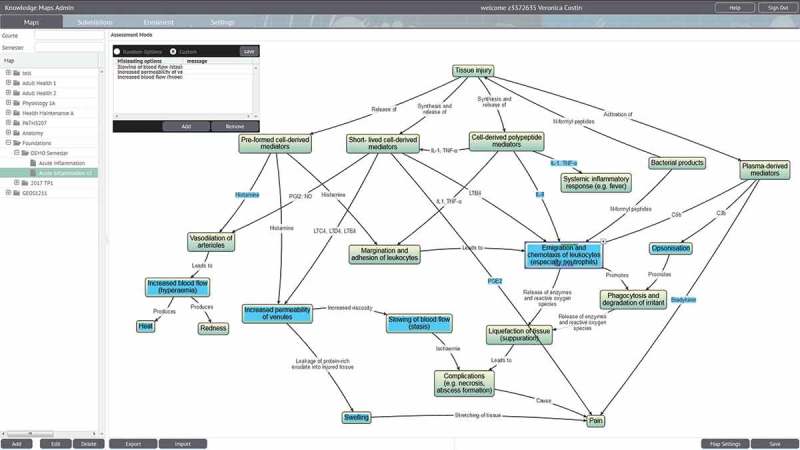
Teacher view of an assessment map with a pop-up box for writing distractors for a node (and specific feedback for each choice is desired).

When creating a map in assessment mode (Figure 1), teachers create or import a concept and/or knowledge map, and then select which concepts or linking phrases will be assessable. For each of these testable nodes (which are highlighted in blue), students are presented with a drop-down list of potential answers to choose from. Teachers can generate their own list of options, akin to the distractors used in MCQs using a pop-up box. Cueing effects can be minimized by presenting students with 15–20 choices, similar to extended matching questions.

The software immediately generates graphical feedback for students, showing incorrect nodes highlighted in red. Unlike the previous versions which gave a percentage score based on the entire map (i.e., including parts of the map that were already given), in assessment mode students receive a score out of 100 based on the percentage of testable nodes that were answered correctly. Teachers can review students’ scores using an Excel spreadsheet generated by the system that contains all attempts for the map, displaying students’ scores for their first attempt, latest attempt, and best attempt.

To evaluate the reliability and validity of Knowledge Maps as a potential assessment tool in medical education, we ran a pilot study in which students’ automated scores on maps were compared to manual grades for a MEQ on the same topic.

### Participants and study design

The study participants were novice medical students enrolled in their first course of a six-year undergraduate medicine program. Online formative assessments are an established part of this course [42]. These formative assessments consist of a mix of MEQs and objective items (including MCQs, labeling of images, ordering events in a chronological sequence). Automated personalized feedback is provided on completion of the formative assessment.

Within the second formative assessment for the 2017 course, we included a MEQ regarding the pathogenetic mechanisms underlying the clinical features of acute inflammation (a major focus of the course). The question read as follows:

This question refers to the scenario in which Johnny, a 10-year old boy, wounded his knee. The wound subsequently became red, hot, swollen and painful, as well as discharging pus. He also developed a fever and felt generally unwell. Explain how those clinical signs developed, based on your understanding of acute inflammation. In your answer, include examples of chemical mediators of this process.

**Figure 2.**
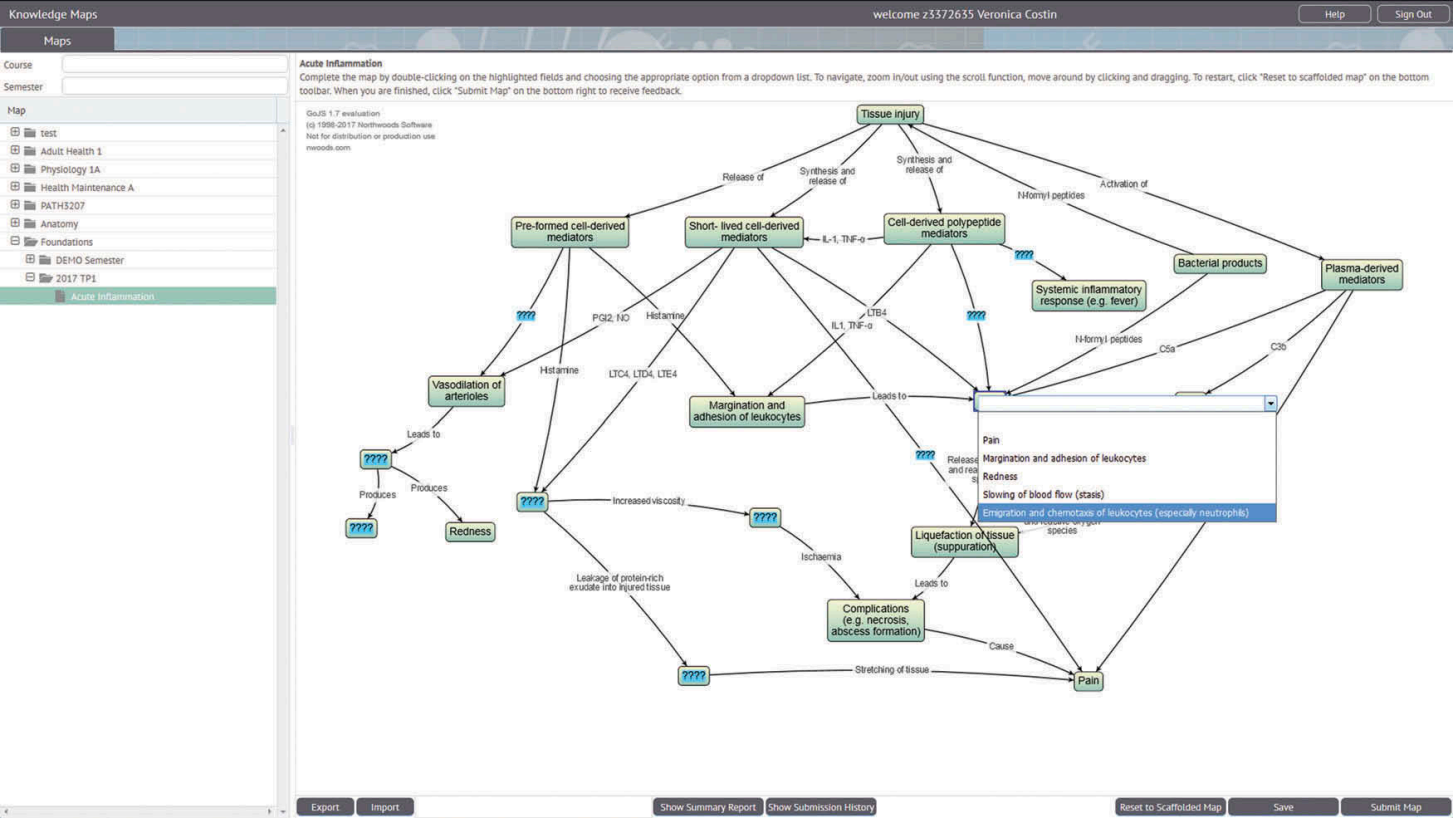
Student view of the acute inflammation map with dropdown list.

Following submission of their answers to the MEQ (and prior to receiving feedback on their response), students were provided with a link to an online knowledge-mapping activity on acute inflammation covering similar concepts to the previous MEQ. The map was revised and edited several times in collaboration with senior members of academic staff in the Department of Pathology and contained 12 testable nodes, each with five options to choose from. No training was provided to students other than the brief instructions at the top of the activity, as seen in Figure 2. Participation was voluntary and students were free to continue with their formative assessment without accessing or completing the map. Those who chose to participate (*n = *137 (50%) out of a cohort of 273 students) attempted the mapping activity (as described earlier) on Knowledge Maps. Students were provided with automated feedback including a percentage score, and then reminded to continue with their formative assessment. Both the formative assessment and the mapping activity were available for one week with an unlimited number of attempts.

Students were also provided with a link to an online questionnaire regarding the perceived utility of the knowledge-mapping tool. 34 students (25% of participants) responded to the questionnaire. Items included students’ perceptions of their understanding of the topic before and after using the knowledge map on a scale of 1–10 (1 = low, 10 = high). Students were also asked to rate different aspects of their experience with the knowledge map on a six-point Likert scale. Open-ended questions enabled students to comment on the strengths and weaknesses of the tool, as well as to provide suggestions for improvements and future use.

As part of the formative assessment, students receive generic feedback on MEQs, including a marking scheme to enable them to evaluate their own responses. For the purposes of this study, answers for the acute inflammation MEQ were independently graded out of 10 by two senior members of academic staff in the Department of Pathology, utilizing the marking scheme provided to students as feedback. Each student’s MEQ score was calculated as the mean of the grades awarded by the two markers, where these grades differed. We performed a correlation between each student’s score for the inflammation MEQ with their percentage score automatically generated by their first attempt at the knowledge-mapping assessment item. Importantly, the markers were blinded to students’ names, scores for the map, and the formative assessment overall, as well as each other’s grades. 103 students who attempted the mapping activity also answered the MEQ.

Approval for this study was obtained from the institutional Human Research Ethics Committee (no. HC15114).

### Statistical analysis

Linear regression analysis was performed to determine the correlation between scores generated by knowledge maps for students’ first attempt and the mean of scores awarded by academic markers. Inter-rater reliability was also determined using Cohen’s kappa. Cronbach’s *α* was calculated to assess the internal consistency of the testable items in the map. The difficulty of each testable node was calculated as the percentage of students who answered correctly. The point biserial correlation coefficient of each testable node was also calculated to determine the level of correlation between identifying the correct answer for an item and each student’s overall score. Students’ perceptions of understanding before and after were compared using Wilcoxon matched-pairs sign ranked test. For Likert scale items, percentage of agreement was calculated (i.e., percentage of ratings greater than or equal to 3.5 out of 6 per item).

## Results

**Figure 3.**
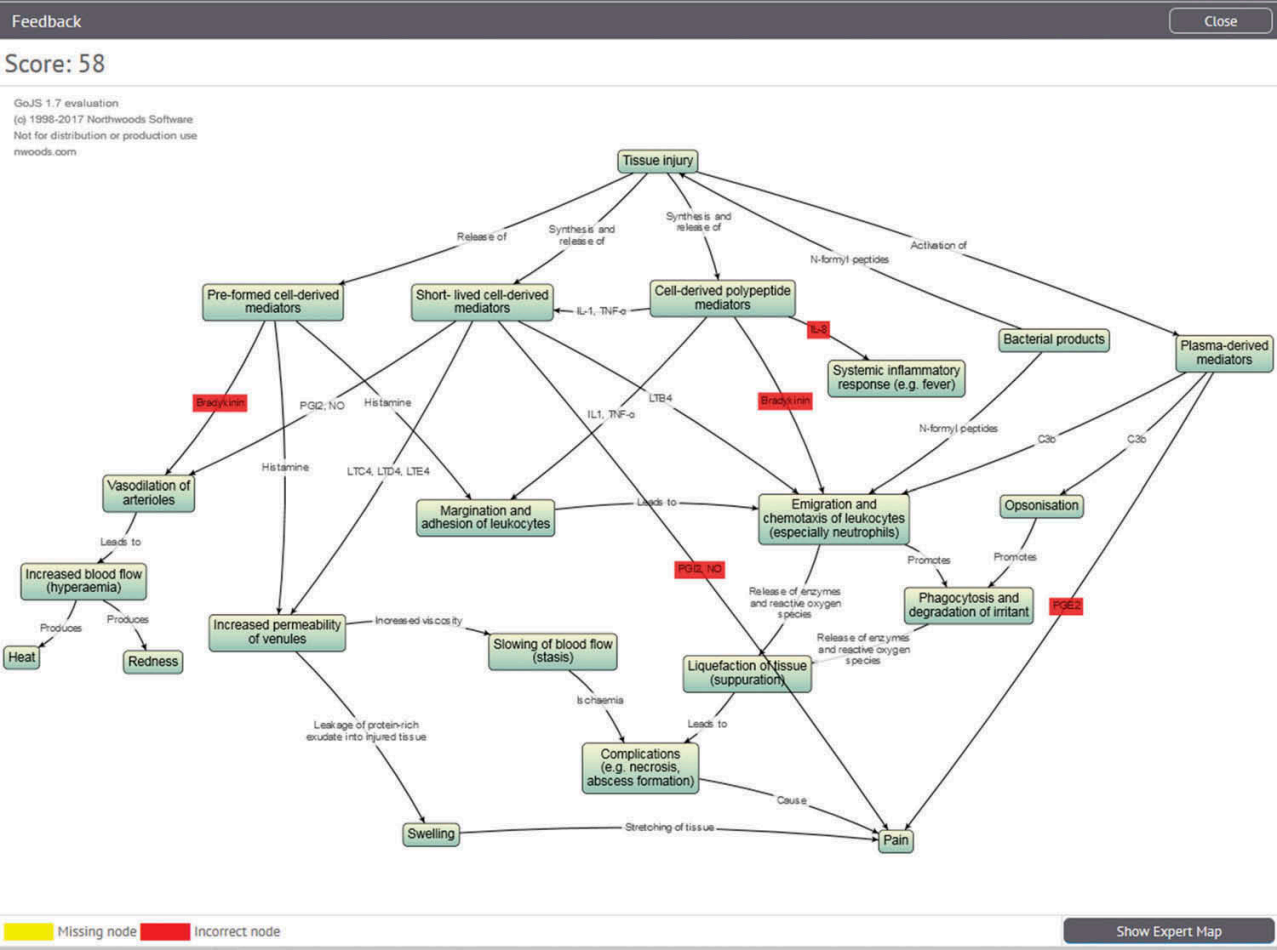
Graphical feedback with incorrect nodes highlighted in red.

Comparison of the map and MEQ responses of low-performing, mid-performing, and high-performing students indicated that similar gaps in understanding were elicited through both assessment items. For example, a low-performing student who received 40% for his MEQ showed limited understanding of the chemical mediators involved in acute inflammation, despite being specifically asked about this. Correspondingly, this student received only 50% for his map, in which he selected incorrect choices for the chemical mediators, but correct choices for the broader processes of acute inflammation (Figure 3).

**Figure 4.**
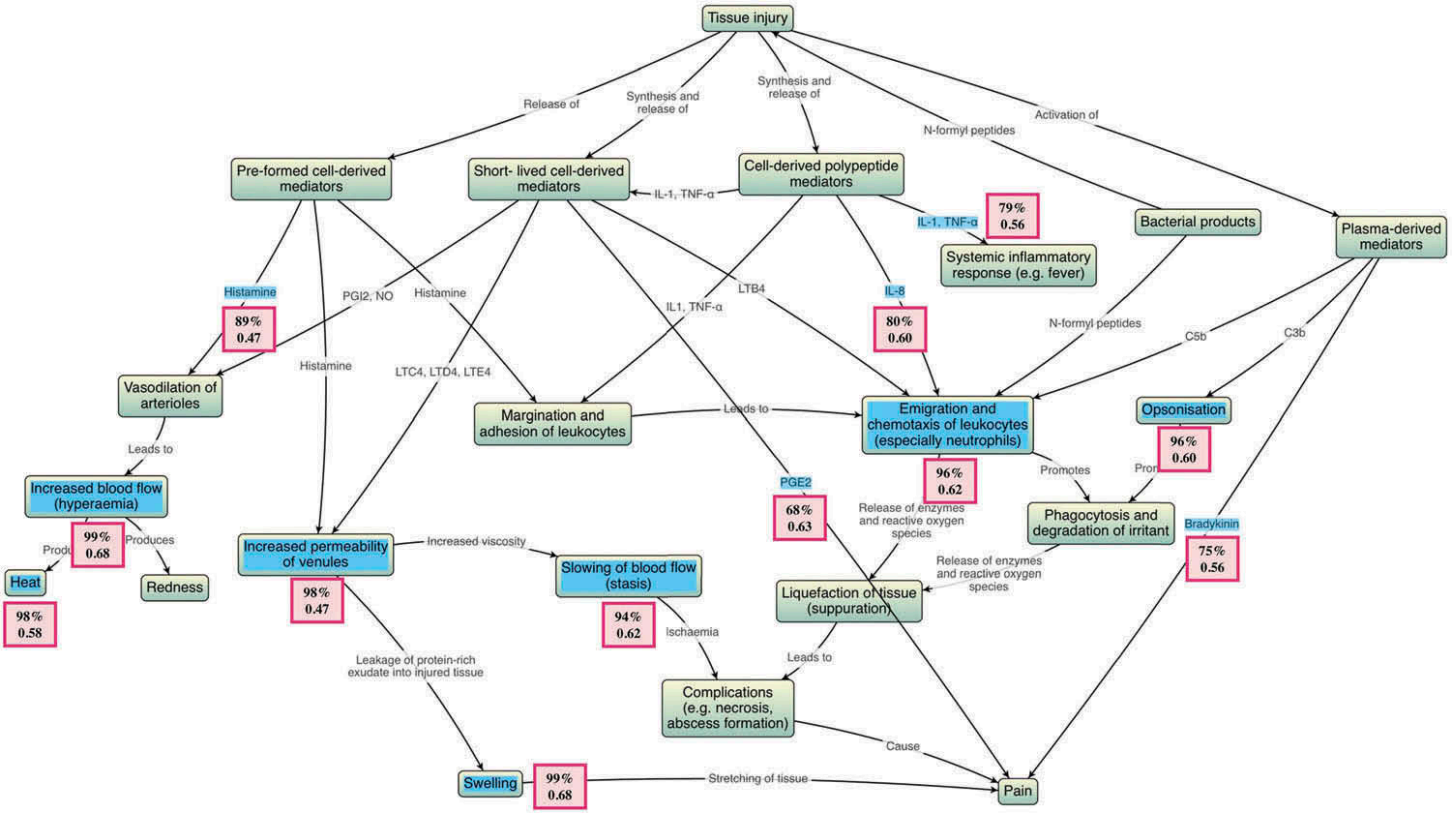
Acute inflammation knowledge map with testable items highlighted in blue; in pink boxes, difficulty (percentage of students who answered correctly) is above and point biserial correlation coefficient is below.

The testable items in the acute inflammation knowledge map were reliable (Cronbach’s *α* = 0.77). The items ranged from moderately easy to very easy – 68.1% to 98% correct (the first number for the corresponding item in Figure 4). Point biserial correlations ranged from 0.469 and 0.684 for testable nodes (the second number for the corresponding item in Figure 4). Overall, there was a significant correlation between map scores and MEQ scores (*r *= 0.481, *p *< 0.001). Inter-rater reliability for MEQ scores was very high (*r *= 0.874, *p *< 0.001).

**Figure 5.**
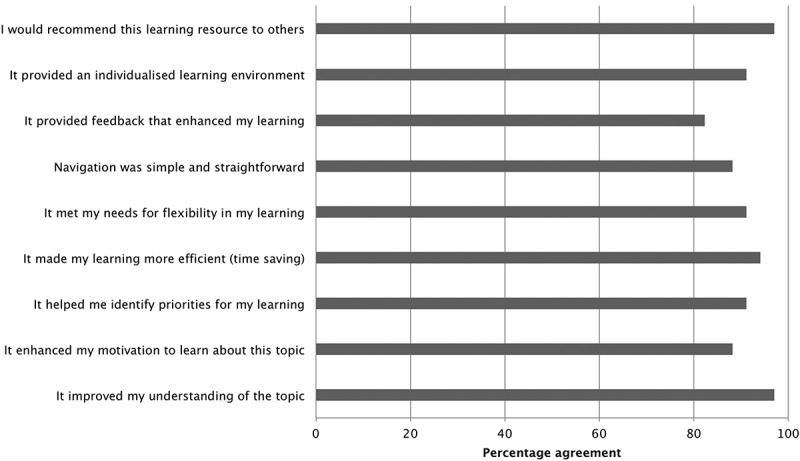
Likert-scale questionnaire responses (percentage agreement [i.e., scores >3 on a scale from 0 to 6]; *n *= 34).

Students reported significantly higher perceptions of understanding the topic of acute inflammation on a scale from 1 to 10 after using the maps (Before: median 7.0, interquartile range (IQR) of 6.0–8.0; After: median 9.0, IQR 8.0–9.0; *p *< 0.001). A summary of responses to Likert scale items on the utility of the knowledge-mapping activity is provided in Figure 5. A large majority of respondents perceived that the mapping tool improved and motivated learning, helped them identify important concepts, was simple to use, as well as being a resource they would recommend to others.

In response to an open-ended item of the questionnaire, ‘Please comment on what you liked most about knowledge maps’, the most commonly identified responses were that the maps were easy to use and that they helped link everything together.

## Discussion

The value of an assessment method can be determined by its validity, reliability, impact on future learning, acceptability, and cost [2,43].

The significant correlation between grades for MEQs and online knowledge maps on the same topic suggests that the mapping activity provided a similar indication of students’ understanding of acute inflammation as the MEQ, without the added marking burden. While the correlation coefficient was not very high, this is unsurprising, given that the two assessment items were not identical, and therefore necessarily examined slightly different aspects of the same topic. Moreover, the integration of concepts from different domains is a fundamental characteristic of concept and/or knowledge maps, implying that this form of assessment could have the advantage of emphasizing a holistic understanding of subject matter. Essentially, students are asked about the relationships between concepts within their broader context and are thus encouraged to understand topics more meaningfully.

Cronbach’s *α* demonstrated high internal consistency across the testable items in the knowledge map (*α* = 0.77). This value is considered a good indicator that students who answer a given item correctly were likely to answer other questions correctly [44]. Even though the majority of students answered most testable nodes correctly, point biserial correlation coefficients across all items were very high, with the lowest value being 0.47 [45–47] . These data indicate that testable map items were highly discriminating between students who performed poorly overall on the map items and students with greater knowledge of the topic.

Our approach was designed to minimize cueing effects, because answering by process of elimination is unlikely to be effective when faced with lists that contain 20 or more options, which is typical for a knowledge map. The number of options is akin to extended matching MCQs, which are known to successfully address cueing concerns. Furthermore, teachers have the advantage of being able to determine different options for each testable node and set up functioning distractors based on known misconceptions.

The effectiveness of such maps as assessment items will of course depend on how well each map is constructed (as well as the options in teacher-generated dropdown lists), but this is no different to any other assessment item. The initial creation of a well-constructed map does take time, effort, and careful consideration [48]. However, an MCQ that addresses key areas of understanding, and discriminates well between low-performing and high-performing students, also takes time to perfect and review [21,23]. While MEQs and other open-ended items may be easier and faster to author, grading responses to these items is highly labor intensive, which is of particular concern if class sizes continue to increase over time [10,49]. The automated grading that is possible with online mapping tasks alleviates the marking burden, and could even be especially beneficial in formative settings by increasing the opportunity for students to receive feedback on their learning. Additional advantages of using knowledge maps in assessment is that the same map can be used to assess different aspects of a topic by testing different nodes, as well as varying the degree of scaffolding provided by altering the number of nodes tested.

Knowledge maps are more user friendly than many existing systems. We have previously shown some of the quantifiable benefits that online testable pathogenesis maps can have for students’ learning [33,34]. However, the process of creating these activities was convoluted and arduous: it required creating a map using CmapTools™, exporting it as an image, removing nodes using image editing software, then finally using these images to create a testable ‘drag and drop’ map using online assessment software such as Questionmark Perception™ (Questionmark Computing Ltd, London, UK). Although the activities and assessments created by knowledge maps appear different, the essential task is the same for students (i.e., fill in blank parts of a scaffolded map from a selection of choices). This software streamlines the process of designing and implementing a mapping activity, thereby making it more acceptable to educators as a teaching and assessment tool. Moreover, such mapping activities can provide students with personalized feedback in real time, thereby saving valuable time for educators.

The perception that knowledge maps is straightforward and easy to use was mirrored in students’ responses to the online questionnaire. The vast majority of respondents perceived the mapping activity very positively. Taken together with previous pilot studies [40], knowledge maps has demonstrated high usability and accessibility. These are essential qualities for any educational tool, particularly if it is to be used for assessment purposes [50,51]. The large loss of subjects for the survey, with a 25% completion rate, is a potential source of bias. However, the survey responses were anonymized and further analysis of which subjects responded was not possible. Additionally, while not a conclusive marker of learning, students did perceive that they understood the subject better after using the map. They also agreed that it helped shape their future study by elucidating areas of understanding and misunderstanding and motivating further study, which are central aims of formative assessment.

Since this trial was conducted, we have introduced a new feature to knowledge maps that provides students with a hint for parts of the map they answered incorrectly. For each option, a different comment can be added, which will be shown to students if they select that option. These hints are viewed by hovering over incorrect nodes. This has the potential to be beneficial when using these maps in formative assessment by helping students understand why their choice was correct or incorrect.

### Limitations

Our participants were restricted to first-year undergraduate medical students. It is possible that more senior students, who might already have their own framework of understanding for a disease process, would find it more difficult to adapt and apply their knowledge to a preconstructed map. Future research into this use of automated knowledge map assessments would be valuable, as would further exploration in its use in other disciplines of study.

This pilot study was limited to a single cohort using one knowledge map on a specific topic. Without further evidence with other topics and cohorts, it is unclear how generalizable and broadly applicable these findings truly are. Nonetheless, these initial results encourage further exploration of the validity and reliability of this tool in comparison with existing methods of assessment [52].

## Conclusion

The outcomes of this study indicate that assessment using online knowledge and/or concept maps with automated grading and feedback could be a useful addition to the current repertoire of assessment items for both formative and summative purposes. Such assessment tools might be beneficial for learning. By their nature, such maps emphasize integration of concepts, and might be useful for assessments in a variety of disciplines.
